# Supplementary material for: Are carbon emissions trading and green financial instruments synergistic? -Comprehensive quantitative research based on content analysis
Source: PLoS One. 2024 Mar 7;19(3):e0298601. doi: 10.1371/journal.pone.0298601 (PMC10919601; doi:10.1371/journal.pone.0298601)
Supplement: S1 Data — (ZIP) [file pone.0298601.s001.zip › Data/地级市Z_F工作报告.zip!/╜¡╦╒/╗┤░▓╩╨/└┤╫╘╣1⁄2╓┌║┼í╛┬φ┐╦╩2╛▌═°í┐_2015.docx]

各位代表：

现在，我代表市人民政府向大会作工作报告，请予审议，并请各位政协委员和其他列席人员提出意见。

一、2014年工作回顾

过去的一年，面对严峻复杂的宏观经济环境和艰巨繁重的改革发展稳定任务，我们始终牢记习近平总书记把周总理家乡建设好的殷切嘱托，按照省委省政府实施“八项工程”、“十项举措”和市委提出的“突破年”总体要求，统筹谋划，积极作为，全力稳增长、调结构、促改革、惠民生、重生态，全市经济社会发展呈现出“稳中有进、质态向好、改革深化、民生改善”的良好态势，圆满完成了市七届人大三次会议确定的各项目标任务，取得了新常态下全面突破的新业绩。主要经济指标增幅继续位居全省前列，列入省统计的18项主要指标中，我市有5项指标增幅位居全省第一，地区生产总值及投资、消费、收入等一批核心指标增幅的位次前移。完成地区生产总值2455.4亿元，同比增长10.9%；公共财政预算收入308.5亿元，增长13.7%；金融机构本外币存贷款余额分别达2036.9亿元、1635.6亿元，增长17.3%和17%；城乡居民人均可支配收入分别达25798元、12010元，同口径增长9.4%、11.6%。“两大目标”建设迈出坚实步伐，中心城市“六大功能”全面提升，全市城镇化率达56.5%，城镇化质量指数位居全国地级及以上城市第55位；以县为单位达到省定2003版全面小康标准，为建设更高水平小康社会奠定了坚实基础。重点领域发展取得突破之效，高速铁路等一批重大工程建设取得突破，引领城乡加快建设和全市跨越发展；土地使用制度等一批重点改革取得突破，改革红利的释放进一步激发了发展活力；航空口岸正式对外开放等一批重点工作取得突破，增添了区域发展的新优势。“突破之年”奋发有为的生动局面，有力带动全市经济社会发展形成全面突破之势。

产业转型升级步伐加快，经济发展实现扩量提质。统筹推进稳增长促转型，经济增长稳中有进，优势特色产业集聚度明显提高，科技创新水平持续提升。经济保持平稳增长。充分发挥投资的关键作用和消费的基础作用，完成规模以上固定资产投资1795.7亿元，增长23%，其中工业投资增长25.6%；实现社会消费品零售总额814.8亿元，增长13.1%；完成进出口总额41亿美元，增长12.2%。强化项目建设的带动作用，14个省级和220个市级重大项目加快建设，开工超亿元工业项目151个，新增规模以上工业企业223户、销售超亿元企业45户，规模以上工业增加值增长12.9%；实施投资超3000万元农业产业项目111个、同比翻了一番，新增销售超亿元龙头企业11户，完成农村水利投入10.6亿元，粮食生产实现十一连增；实施投资超亿元经营性三产项目158个，服务业增加值突破千亿元；建筑业产值达1226亿元。产业结构不断优化。“4+2”优势特色产业产值占规模以上工业产值比重达55.2%，提高6.1个百分点。电子信息产业产值超过900亿元，盐化新材料产业创成省级特色产业集群，食品产业产值位居全省前列，特钢重点企业加快转型发展，获批省新能源汽车先进制造业基地。新兴服务业蓬勃发展，新增规模以上生产型电商企业超过200户，淮安软件园成为省级电子商务示范基地，获批国家4A级景区2个、省级旅游度假区1个，服务业增加值占比提高1个百分点。现代农业加速发展，获批1个省级现代渔业产业园区，新创2个中国驰名商标，新增家庭农场数全省第二，农业现代化工程发展指数和综合机械化水平苏北第一。创新能力明显增强。全社会研发投入占GDP比重超过1.6%，新增国家高新技术企业76户、省级企业创新平台30个，专利申请量增幅全省第一，高新技术产业产值占规模以上工业产值比重提高1个百分点。落户知名高校院所研究机构15家，创成苏北第二家国家级大学科技园，建成中国留学服务中心淮安分中心和江北首家海创智库科技服务中心。设立淮上英才创投基金，引进国家“千人计划”专家20人、省“双创计划”人才18人。

改革开放互动并进，发展动力活力不断增强。坚持先行先试，积极向上争取，土地使用制度、“多规合一”等8项改革纳入国家试点，新型城镇化综合配套等15项改革列入省级试点，形成了具有淮安特色的改革体系；获批首家全国台资企业产业转移集聚服务示范区，进一步彰显了淮安对外开放的品牌特色。重点领域改革加快推进。行政审批制度改革取得重要进展，成为国家首批社会管理和公共服务综合标准化试点，建成全国一流的政务服务中心，市级行政审批事项由119项精简至42项，有效激发了市场主体活力，新增私营企业和个体工商户分别增长28.3%和52.1%，成为省创业型城市。金融改革不断深化，今世缘酒业实现我市企业境内主板上市零的突破，引进银行机构2家，组建科技支行3家，设立中小微企业低息统贷平台和应急过桥资金，村级金融综合服务站覆盖面达75%。土地使用制度改革全面启动，开展15个重点功能片区规划编制，稳步推进农村土地承包经营权确权颁证和集体资产股份制改革试点工作。新型城镇化综合配套改革扎实推进，出台加快新型城镇化的若干意见，推进“多规合一”改革，强化科学规划引领，加快构建“一区两片四轴”市域空间格局，优化形成“1047”小城镇规划体系，行政区划调整、扩权强镇等改革有序推进。对外开放质态有效提升。创成省级利用外资转型发展示范区，新批投资超9000万美元项目13个，注册外资实际到账12亿美元，分别位居苏北第一、第二。引进英国信诺医疗等项目，服务业利用外资占比提高15个百分点。创立苏北首家外贸综合服务企业，新增外贸超千万美元企业15户。新增国际友城、友好交流城市6个。台资集聚高地加速崛起。全面实施项目三年倍增、交流平台扩展、101%服务升级“三大计划”，引进投资10亿美元的臻鼎科技等一批重大项目。成立大陆首家台商学院，成功举办第九届台商论坛，连续三年被评为台商投资“极力推荐城市”。重大项目招引取得突破。新引进投资超亿元内资项目216个，其中超10亿元项目25个，超50亿元的重特大项目5个。

重点工程建设成效显著，新型城镇化全面推进。“十项重点工程”建设取得突破，“一县一策、一县一题”专项扶持扎实推进，有力支撑了中心城市建设和县域经济发展。中心城市功能进一步完善。投资1007亿元，实施910个重点项目，“一廊四区多片”功能特色逐步显现。连淮扬镇铁路启动建设，徐宿淮盐铁路即将开工，我市在长三角乃至全国的综合交通枢纽地位得以确立，淮安人民正跑步进入高铁时代；里运河文化长廊建设全线推进，清江浦景区、状元府第等项目建成开放；古淮河·西游文化旅游区建设步伐加快，现代有轨电车1号线全线铺轨，白马湖森林公园和湿地公园建设初见成效，市体育中心建成运营，智慧产业谷一期工程主体竣工，金融中心开工建设，西南化工区居民搬迁基本完成。全面提升城市夜景亮化，基本建成数字城市地理空间框架，积极导入城市形象识别系统，加强城市长效管理，全国文明城市创建获得提名资格。县域经济发展后劲不断增强。涟水电子电气产业园升格为省级特色产业园区，洪泽获批建设国家现代农业示范区，盱眙旅游业成为县域经济重要增长点，金湖开发区位列全省开发区综合评价第一版块，清河现代服务业加速高端化发展，清浦生物医药产业优势凸显，淮安国信工业园特色产业加速集聚，淮阴规模以上工业产值率先突破千亿元。镇村建设水平不断提高。在加快四座县城建设的同时，着力推进10个重点中心镇建设。新改建农村公路588公里、桥梁82座，镇村公交开通率达35.6%。完成10773个村庄环境整治，施河镇创成苏北唯一的全国卫生镇，老子山镇龟山村入选中国传统村落名录。生态文明建设扎实推进。在苏北率先通过省级生态市考核验收，成为全国生态文明先行示范区和水生态文明城市建设试点市，获批国家新能源示范城市。铁腕整治大气污染，秸秆综合利用和企业减排工作全省领先，PM2.5浓度降幅全省第二，全年雾霾天数下降明显，既有效改善了全市空气质量，又为全省大气污染防治作出了积极贡献。

民生保障水平持续提高，公共服务能力进一步增强。坚持以兴办惠民实事为抓手，统筹推进公共服务、社会保障和安全稳定，民生优先的工作导向更加鲜明，群众的幸福感和满意度逐步提升。十件为民实事全面完成。投入40.1亿元，兴办了一批事关群众切身利益的民生项目，整治改造36个老旧小区、惠及2万多户居民，新增区域供水乡镇33个，解决了45.35万农村人口饮水安全问题，新扩建省优质幼儿园44所，提升100所农村中小学教育装备水平；解决了一批群众反映的突出问题，完成淮海南北路改造等道路通达工程，改造升级市区农贸市场12个，新建公共停车位3726个、绿色小客厅15个、公厕104座；改善了一批困难群体的生活条件，新增农村脱贫人口16万人，为6154户残疾人家庭适配辅助器具。社会事业协调发展。以市为单位通过县域义务教育发展基本均衡国家督导认定，获省政府支持筹建综合性淮安大学，获批成立江苏护理职业学院。建成淮安协和医院、口腔医院，启动建设市儿童医院、眼科医院，市第二人民医院创成“三甲”综合性医院。大运河成功入选世界文化遗产名录，新增2项国家非物质文化遗产，创成一批省级公共文化服务体系示范区，版权登记总量全省第四。实现全国计划生育优质服务先进县区全覆盖。成功举办第十八届省运会，不仅赛会成绩创历史最佳，更以焕然一新的城市面貌和周密高效的赛事组织，进一步提升了淮安的影响力和美誉度。社会保障水平逐步提高。新增城镇就业再就业10.1万人，城镇登记失业率降至2.2%。城乡基本社会保险覆盖率持续巩固，基础养老金标准提高至每人每月90元，城乡低保标准分别提高到450元和330元。全面完成经济适用房和公共租赁房建设任务，惠及困难群众2000多户，发放公积金贷款18.9亿元，成为全国六个共有产权住房改革试点城市之一。开通阳光慈善网，新建20个区域性养老服务中心，改扩建47所农村敬老院 金庸小说。社会治理创新不断加强。推进平安淮安、法治淮安建设，全面推行“政社互动”社区治理模式，“阳光信访”升级版在全国推广，安全生产形势平稳趋好，食品药品安全监管力度不断加大。

作风建设深入推进，行政服务效能明显提高。深入开展党的群众路线教育实践活动，大力弘扬“包容天下，崛起江淮”的新时期淮安精神，风清气正、干事创业的氛围更加浓厚，服务发展、服务群众的水平进一步提升。坚持勤政为民。积极开展“微调研”，开通“代表委员直通车”，组织多形式的走访、座谈活动，面对面听取群众的意见和建议。针对群众反映的热点难点问题，深入开展专项整治行动，对19项重点内容、101个整改项目，以“钉钉子”精神狠抓整改落实，解决了一批入学、就医、出行、饮水安全等方面的突出问题，打通了联系服务群众的“最后一公里”。坚持务实高效。积极顺应群众对政府工作求新求快求实的热切期待，着力提高行政服务效能，精简会议文件，集中时间和精力抓好落实。实行重大项目挂钩帮扶机制，完善公开承诺、首问负责等制度，出台行政问责办法，形成了真抓实干的鲜明导向。坚持清正廉洁。认真落实党风廉政建设责任制，严格履行“一岗双责”，加大反腐倡廉力度。按照中央八项规定精神，出台厉行节约反对浪费等规章制度，严格规范公务接待，严把因公出国出境会审关，严控“三公”经费支出；加强对重点领域、关键部门和专项资金的审计监督，进一步强化了制度的刚性约束。各项工作协调推进。深化军民融合发展，在支持地方建设中发挥积极作用。全面完成年度援疆任务，援疆工作走在新疆生产建设兵团和全省前列。统计、物价、国资、质监、海关、检验检疫、供销等部门服务经济发展能力进一步增强，广播电视、民防、机关事务、气象、地震、邮政、仲裁、档案、地方志、民族宗教、老龄、妇女儿童、青少年、关心下一代、红十字等事业加快发展。

回顾过去一年的工作，在省委省政府和市委的正确领导下，全市上下团结一心、创新实干，战胜了异常艰巨的困难挑战，形成了全面突破的发展态势。成绩来之不易，经验尤为可贵。我们深切体会到，实现淮安科学跨越发展，必须认清形势、把握大势。只有因势利导、统筹谋划，才能保持定力、应对有度，进一步抢抓机遇、打牢基础、行稳致远。必须勇于探索、改革创新。只有先行先试、开拓进取，才能内挖潜力、外聚要素，进一步增强动力、激发活力、争创优势。必须敢于担当、攻坚克难。只有矢志不渝、义无反顾，才能迎难而上、负重拼搏，进一步排除障碍、破解瓶颈、再上台阶。必须注重团结、加强协作。只有密切配合、协调一致，才能攥紧拳头、形成合力，进一步汇集智慧、聚合能量、跨越争先。同时，我们更加深刻地感受到，健康的政治生态、和谐的社会氛围、包容的人文环境，不仅坚定了我们推动发展的信心和决心，也增强了我们战胜困难的勇气和力量，让我们时刻牢记使命，丝毫不敢懈怠。在此，我谨代表市人民政府，向全市广大干部群众，向全体人大代表、政协委员，向各民主党派、工商联、无党派人士、各人民团体和离退休老同志，向各驻淮单位及部队指战员，向在淮的全体客商和建设者，向所有关心支持淮安发展的海内外朋友，表示衷心的感谢并致以崇高的敬意!

同时，我们清醒地认识到，全市经济社会发展中还存在一些困难和矛盾，主要是：稳增长压力较大，企业生产经营困难较多，部分经济指标增幅有所回落；调结构任务艰巨，战略性新兴产业比重还比较低，科技创新支撑作用还不够强；促改革难点增多，一些重点领域和关键环节改革力度还不够大；惠民生还有差距，在统筹解决群众的新期盼方面还存在薄弱环节；转职能还不够到位，政府依法行政能力有待提高。对这些问题，我们将予以高度重视，采取有效措施，切实加以解决。二、2015年主要任务

今年是“十二五”规划的收官之年，是全面深化改革的关键之年，是全面推进依法治国的开局之年，也是淮安新时期“三步走”战略的“上水平”之年。面对经济发展新常态，我们必须立足淮安实际，既要积极适应、顺势而为，更要把握机遇、乘势而上。正确面对新常态下出现的新挑战，经济下行压力较大，增加投资、扩大消费的任务还很艰巨；地方政府性债务管理和招商优惠政策的严格规范，使得融资、引资的难度明显加大；生产要素价格总体上升以及资源环境约束趋紧，后发地区的比较优势正在分化。这对我们的发展理念和工作方法都提出了新的要求。准确把握新常态下孕育的新机遇，我国发展仍处于可以大有作为的重要战略机遇期，特别是国家各项改革不断深化、精准调控持续发力，将为我们释放更多的政策红利；“一带一路”、长江经济带等重大战略的深入实施，省委省政府打造沿运河城镇轴，区域互补、跨江融合、南北联动战略部署的不断推进，将为我市拓展更大的发展空间。充分认识新常态下淮安的新优势，特殊的发展阶段蕴藏着巨大的成长优势，完备的综合交通体系凸显出更佳的区位优势，崛起的台资高地呈现出独特的开放优势，良好的生态环境展现出持续的竞争优势，包容的特色文化彰显出和谐的人文优势。这些优势的叠加，奠定了淮安跨越发展的坚实基础，使我们更加充满信心。只要把握发展大势、积极主动作为，就一定能在新常态下取得新业绩、迈上新台阶。

今年政府工作的总体思路是：全面贯彻落实党的十八大、十八届三中、四中全会和中央经济工作会议、省委十二届九次全会精神，紧紧围绕市第六次党代会第四次会议部署，积极适应经济发展新常态，准确把握稳中求进总基调，始终贯穿“上水平”工作主线，围绕“两大目标”，聚焦“五大突破”，坚持以提高经济发展质量和效益为中心，以改革开放和科技创新为动力，以优化投资和扩大消费为支撑，以改善民生和生态文明建设为根本，努力开创全市经济社会平稳健康发展的新局面。

今年主要预期目标是：地区生产总值增长10%左右，公共财政预算收入增长11%，规模以上固定资产投资增长18%，社会消费品零售总额增长13%左右，注册外资实际到账增长5%，城乡居民人均可支配收入分别增长9%和11%，城镇登记失业率控制在4%以内，居民消费价格指数不超过全省平均水平，节能减排完成省定任务。这些指标是经过综合研判、慎重权衡确定的，既积极顺应了经济发展新常态的新要求，也充分考虑了淮安发展新阶段的新特征。部分指标增幅有所下调，主要是为调整经济结构和深化改革腾出更大空间，并着重在经济发展效益、城乡建设质量、人民生活福祉等重要方面实现上水平。

做好今年工作，我们要以习近平总书记视察江苏时的重要讲话精神为根本遵循，牢牢把握“迈上新台阶，建设新江苏”的新要求，不断丰富“把周总理家乡建设好”的新内涵，着力打造经济发展、城乡建设、和谐构建“三个升级版”，努力探索新常态下科学跨越发展的新路径。围绕上述目标要求，着重抓好以下六个方面工作：

(一)坚持量质并举稳增长，着力提升经济运行质态。强化科技创新支撑，加快发展先进制造业和现代服务业，积极构建现代产业体系，推动经济扩量提质，加快产业转型发展。

高端化发展先进制造业。实施“四千”提升计划，实现规模以上工业增加值1300亿元，增长12%左右。着力打造千亿元产业，加快实施中兴4G智慧产业园、纳沛斯半导体等重点项目，推动电子信息产业产值率先突破千亿元；加快建设金岭氯碱产业园等龙头项目，促进盐化新材料产业向精细化、高端化发展；食品产业重点做大规模、做响品牌，特钢产业加快与装备制造业的融合发展，高端装备产业加速向智能化、成套化方向迈进，新能源汽车产业力争实现整车生产的历史性突破。围绕实现千亿元投资，重抓30个以上投资超10亿元的重大项目，新开工超亿元工业项目150个，新竣工设备投资超千万元项目100个，规模以上工业投资增长19%。积极推动千企技改，实施投资超3000万元重点技改项目100项，技改企业超过1000户。切实强化千企帮扶，落实“一企一策”长效机制，重点帮助1000户成长型企业做好要素保障和市场开拓等工作，新增规模以上企业150户、销售超亿元企业35户。完成省级开发区空间布局整合和产业规划修编，扶持特色产业基地建设，力争县区百亿元支柱产业培育工程取得突破。

特色化提升现代服务业。突出规划引领、项目支撑、政策扶持和机制创新，做强物流、金融、旅游、商贸、医疗卫生五大基础服务业，做大电子商务、健康养生、文化创意三大新兴服务业，加快构建“5+3”现代服务业体系，成为新常态下淮安经济发展的新引擎。大力发展旅游业，重点围绕“一河一湖一园一线”做文章，加快里运河文化长廊、白马湖生态旅游度假区、西游记文化体验园建设，整合推出以乡村旅游为特色的自驾游线路，使旅游业尽快成为淮安的支柱产业。优先发展物流业，依托综合交通枢纽优势，加快各类现代物流园建设，大力发展产业物流、快递物流、港口物流、保税物流等业态，着力打造区域性商品集散、商贸商务和物流中心。重点发展电子商务，加快建设网上购物、网上交易、在线支付等综合网络信息服务平台，推动实体经济与网络经济深度融合。突破发展健康养生业，发挥我市生态良好、气候宜人、医疗体系健全等优势，积极打造健康养生产业链。

协同化推进科技创新。积极创建国家创新型试点城市，全社会研发投入占GDP比重达1.7%，高新技术产业产值占工业产值比重超过26%。提升企业创新能力，启动实施创新型企业梯队培育、知识产权战略推进“三年行动计划”，积极鼓励企业增加投入，加大招才引智、科技研发力度，新增国家高新技术企业40户。强化创新载体平台建设，加快科技人才创业特区和科技广场建设，省级以上各类园区在孵企业达到500户。加大协同创新力度，引导驻淮高校院所与企业开展多种形式的产学研合作，建成国家“千人计划”盐化新材料产业研究院，新建一批协同创新基地、企业科技服务站和校企联盟，引进国家“千人计划”专家20人。支持“淮上英才”创投基金扩量增效，扶持一批优质科技企业上市，探索建立专利成果转化交易平台，努力为所有想创新能创新者提供机会、搭建舞台。

(二)坚持改革开放添活力，着力提升要素集聚能力。以深化改革促进对外开放，以扩大开放倒逼各项改革，进一步集聚优质要素，激发内在活力。

抓住重点环节深化改革。抢抓国家和省试点机遇，以重点领域改革为突破口，统筹推进各项改革。进一步深化行政管理体制改革，全面梳理并公布行政权力目录清单，实行最严格的行政许可项目准入；按照“大厅之外无审批”的要求，加强市县乡村四级政务服务体系建设，全面提升行政服务效能；深化市场准入与监管制度改革，进一步降低市场准入门槛、放宽经营场所限制，大力推进全民创业、淮商崛起，让广大创业者有更多的出彩机会。加快土地使用制度改革，探索片区规划、地随人走的土地管理新路径，推进重点产业园区、重点中心镇的功能片区规划编制和土地整治，优化空间布局，保障城乡发展；稳步推进不动产统一登记，有序推动农村集体土地承包经营权流转，积极开展集体建设用地制度改革试点，进一步完善严格保护耕地、节约集约利用土地的激励机制。加大金融改革力度，完善投融资体制，推广政府与社会资本合作的PPP融资模式，推动政府融资平台转型升级；加快企业上市步伐，加大银行机构引进力度，开展农村土地承包经营权抵押贷款业务试点，切实优化金融生态环境。全面推进新型城镇化综合配套改革，加快出台户籍管理、社保并轨等政策措施，放开城镇落户限制，实行社会保障一卡通，让农民进得了城、扎得下根、生活得好；深入推进“多规合一”改革，启动城市总体规划修编，稳妥推进区划调整，扩大重点中心镇扩权强镇试点，打造一批新型城镇化试验区、城乡统筹发展示范区。统筹推进国有企业、医疗卫生、文化科技等领域的改革，不断丰富具有淮安特色的改革体系。

依托载体建设扩大开放。尽快实现淮台直航，适时拓展国际航线，打造淮安对外交流合作的“空中走廊”。放大综合保税区平台功能，加强空港产业园建设，建成进口商品展示展销中心。扶持市级进口代理和外贸综合服务平台发展，帮助企业开拓国际市场，稳定外贸增长势头。加快重点国别园区建设，落户一批外资项目。深入推进台资高地建设“三大计划”，加强台资经济研究，促进台资企业转型升级，着力打造好国家级台资企业产业转移集聚服务示范区这一“金字招牌”。

突出产业集聚招引项目。积极做好新常态下的招商工作，及时调整思路、规范行为、提高本领，培育专业化的招商人才队伍，打造101%服务升级版，不断增创服务高地新优势。围绕培育壮大优势特色产业，引进投资超亿元内资项目210个，力争超10亿元项目30个，并在超50亿元重特大项目引进上取得新的突破。切实提高外资招商成效，引进投资超3000万美元项目30个以上。

(三)坚持优化布局促统筹，着力提升新型城镇化发展质量。强化科学规划引领，注重空间布局优化，加快中心城市引领下的特色城镇化进程。

增强中心城市功能。围绕提升中心城市“六大功能”，投资741亿元，实施761个项目，全面增强中心城市综合承载力和辐射带动力。强力推动“十项重点工程”，里运河文化长廊开工建设漕运古城等一批骨干项目，建成清江浦1415街区、萧湖景区等节点工程，实现慢行系统全线贯通；开工建设西游乐园，建成龙宫大白鲸嬉水世界；建成开放白马湖森林公园等重点景区；智慧产业谷一期工程投入使用，国家级大学科技园主体功能区建成运营；加快建设金融中心一期工程，签约入驻一批金融机构和企业总部；淮安综合大市场加快转型升级，争创省级电子商务示范基地和国家级商贸示范功能区。加快“一廊四区多片”建设，以里运河文化长廊为主轴，不断提升淮海中心商业区、水渡口中央商务区、河下文旅开发区三大商圈集聚能力，加快生态新城国家级绿色低碳示范核心区建设；强化规划调控作用，统筹推进清河新区、清浦新南城、淮阴新北城、南部工业新城等片区建设，提升组团发展水平。规划建设城市快速交通系统，建成运营现代有轨电车1号线，开工建设淮安机场连接线，启动实施淮海南路京杭大运河桥等一批工程，尽快实现中心城区公共交通与航空、高速铁路、高速公路“零换乘”。推进城市环境综合整治，强化城管集中综合执法，加强乱停乱放、占道经营等交通秩序整顿，加快25个棚户区改造，实施古黄河滨水景观提升工程，推动智慧城市建设，确保通过国家卫生城市复查，为创建全国文明城市打下坚实基础。

推进综合交通枢纽建设。围绕构建以高速铁路为骨干、国际空港为优势、高等级公路为支撑、内河航运为特色的现代综合交通体系，加快建设连淮扬镇铁路，开工建设徐宿淮盐铁路，同步推进综合客运枢纽和连接线等规划建设，积极推动宁淮城际铁路列入“十三五”规划；实施机场二期扩建工程，完善航空口岸对外开放设施，加快打造国际化空港；实施503省道、235国道等干线公路境内工程，不断完善高等级公路网络；大力推进淮河出海航道整治等重大工程，加快建设以淮安港为核心的通江达海内河航运体系。

落实“一县一题”帮扶措施。坚持上下联动、精准发力，帮助县区解决一批事关长远发展的重大课题。支持涟水高沟镇打造现代小城市，争创省级新型城镇化示范区。扶持洪泽建设江苏食品科技产业园，打造全国农业产业化示范基地。引导盱眙凹土产业转型升级，创建国家火炬计划产业基地。加快金湖尧文化旅游产业园建设，力争创成国家4A级景区。推动清河重点片区旧城改造，打造省级特色街区。改造提升清浦文庙功能片区，彰显历史文化特色。扶持淮安电子商务现代物流产业升级发展，创成省级电子商务示范基地。推进淮阴“一区五园”产城融合发展，积极申创国家高新技术产业开发区。

突破重点城镇发展。着力提升四座县城功能，加快盱眙撤县设市步伐，推动涟水综合功能提升，促进淮洪一体化建设，支持金湖构建“一主四辅”格局。大力培育10个重点中心镇，强化片区规划引领，统筹推进“三集中”，落实关键措施，实施功能性项目，尽快将高沟、马坝、车桥打造成县域副中心、现代小城市。因地制宜推进47个特色镇建设，加快建设一批宜居宜业的小城镇。围绕产镇融合发展，推动乡镇工业集中区改造升级，打造10个以上市级工业园区和一批特色产业园区。

(四)坚持健全体系转方式，着力提升现代农业发展水平。加强项目建设、科技投入、品牌引领和机制创新，积极构建现代农业的产业体系、经营体系和保障体系。

链式发展优势产业。按照产业链、价值链等现代产业组织方式，推动农业“接二连三”发展。坚持引进和培育并举，推动龙头企业和重点项目加速向优势产业聚集、向重点区域聚合、向特色品牌聚拢。主攻农产品精深加工、现代物流和农业综合体项目招商，引进实施投资超3000万元农产品加工项目35个。加快农业产业化项目建设，推动肉制品、食用菌等产业链向上下游延伸，支持重点龙头企业按照国家级、省级标准提档升级，新增销售超亿元龙头企业10户。加强农产品品牌市场化运作，努力以品牌开拓市场空间，以市场彰显品牌价值。

精致打造园区载体。整合使用项目资金，支持各类经营主体进驻园区，建设一批集生产生活生态功能于一体的现代农业产业基地。加快建设洪泽国家现代农业示范区，以中心城市为依托打造现代都市农业圈，以白马湖国家农业公园为龙头打造淮金路沿线现代农业产业带。淮安国家农业科技园区通过验收，10个省级农业园区基本实现物联网技术、质量追溯监管平台全覆盖，建成3个精品农业园区，创成1个省级农业产业园区。

多元构建经营体系。大力培育农业经营新型主体，推进家庭经营、集体经营、合作经营等协同发展。加强农业职业技术培训，加快培养有文化、懂技术、会经营的新型农民。引导专业大户向家庭农场转型，鼓励能人大户、农村集体经济组织牵头组建土地股份合作社，积极开展联耕、联种、联管、联营试点，新培育家庭农场400个、土地股份合作社100个、市级以上示范合作社100个。依托互联网拓展农产品营销渠道，推动农业生产与市场需求的有效对接。

全面提高保障水平。加强农业基础设施建设，完成水利投入30亿元，基本建成入江水道整治等治淮骨干工程，新建高标准农田28万亩，提高粮食现代仓储水平。健全农业产学研一体化机制，加大农技推广力度，加强政策性农业保险和气象为农服务，推动农业生产全程机械化，努力提高劳动生产率、资源利用率和土地产出率。

(五)坚持绿色发展增优势，着力提升城乡人居环境。严守生态红线和环保底线，努力以绿水秉赋打造城市特色，以生态环境赢得竞争优势。

全力争创国家生态市。严格对照标准，加大投入，打牢基础，相关县区通过国家生态县区考核验收，力争在苏北率先创成国家生态市，加快建设国家生态文明先行示范区。强化污水管网建设，规范污水处理厂运行，提高排放达标率 完美世界小说。以森林公园、自然湿地和农田林网为载体，加快构建城乡绿色生态系统。扎实推进国家低碳试点城市建设，实行温室气体排放清单制度，加大落后产能淘汰力度，构建跨区域碳交易体系，完善生态补偿机制，确保单位GDP二氧化碳排放量比“十一五”末降低20%，努力让全市人民分享更多的“绿色福利”。

铁腕整治环境污染。严格落实大气污染防治各项措施，深入开展重点企业脱硫脱硝除尘改造、挥发性有机物治理、燃煤锅炉整治、老旧机动车淘汰等专项整治行动，强化建筑工地绿色施工管理，确保空气质量继续保持全省前列。抓好冶金、化工等重点耗能行业的节能减排，重点企业实现节能10万吨标准煤。加强水资源保护和水环境治理，做好全国水生态文明城市试点工作，实施淮河流域水污染防治工程，建设市固体废弃物填埋场，工业废水排放达标率超过95%，饮用水源地水质达标率保持100%。

加快建设美丽乡村。完成村庄布局规划编制，强化集体建设用地管理，坚决遏制农村违规建房。推进农村建设田园化，强化分类指导，编制修建性详规，引导建设100个以上富有地域特色和江淮水乡风貌的特色村庄。推进农民居住社区化，按照新型社区标准，加快基础设施和公共服务向农村延伸，改造提升农村公路300公里、桥梁40座，着力打造300个左右重点村庄，带动村庄撤并和空心村整治。推进村庄环境整洁化，整治11862个村庄环境，全面完成省五年行动计划任务，切实让人们看得见山水、记得住乡愁。(六)坚持兴办实事惠民生，着力提升群众幸福指数。顺应人民群众过上更好生活的期盼，持续加大民生投入，不断改善公共服务，切实用各级干部的奉献赢得广大群众的“点赞”。

认真办好十件实事。围绕人民群众关心的热点难点问题，投入47.2亿元兴办民生十件实事。一是优化市民出行环境，持续实施市区道路畅通工程、公用停车场建设等项目。二是改善农村生产生活条件，推进农村道路升级、户厕改造等项目，新增区域供水乡镇40个，基本实现区域供水全覆盖。三是保障困难群体住房，新开工或收储公共租赁房1130套。四是改善基层办学条件，实施中小学校舍安全工程，按照省一类标准提升80所农村中小学教育装备水平。五是升级改造主城区农贸市场17个。六是优化创业就业服务，新增就业6万人，开展创业培训2万人。七是关心帮助特困群体，城乡低保标准分别提高到490元和370元。八是整治提升城区环境，改造老旧小区69个，新建城市绿色小客厅10个，整治背街小巷52条、排涝河道10条。九是完善便民服务设施，实现行政村综合便民服务点全覆盖。十是继续开展送文化下乡下基层活动。

加快发展社会事业。积极推进教育现代化，加快建设全国义务教育学校管理标准实验区、省学前教育改革发展示范区，推动教育优质均衡化发展，完成淮安开放大学主体建设，积极筹建综合性淮安大学，着力构建产教深度融合的现代职业教育体系。健全现代医疗服务、卫生监管等体系，启动建设市公共卫生服务中心，建成淮安眼科医院和儿童医院一期工程，实施英国信诺医疗合作项目。大力推进文化强市建设，加强历史文化挖掘保护，强化大运河淮安段重要遗产点的保护提升，筹办好清江浦开埠600周年纪念活动，增强运河之都的文化影响力；实施文化惠民工程，深入创建省级公共文化服务体系示范区，加快建设主题博物馆群，建成淮安广播电视塔，构建市县乡村四级文化设施网络；推进文化产业发展，加快打造省级乃至国家级文化产业园区，鼓励重点文化企业兼并重组，创作更多的文艺精品，提升文化产业竞争力。大力开展全民健身运动，推动公共体育服务示范区建设。积极争创全国幸福家庭活动示范市，促进人口协调发展。全民科学素质提升工程通过国家验收。全力争创全国双拥模范城“四连冠”。

不断完善社会保障体系。全面落实城乡一体的就业创业扶持政策，多渠道促进城乡居民增收。实施特困群体养老保险助保工程，继续推动被征地农民参加养老保险，做好机关事业单位人员与城镇职工养老保险并轨工作。完善居民医保大病保险政策，推进新农合与城镇居民基本医疗保险制度融合。加大困境儿童、残疾人等救助力度，完成农村敬老院建设管理提升工程，为特殊困难群体购买养老服务，打造信息化社会化养老服务平台。大力整治“餐桌污染”，切实保障食品药品安全。扎实推进刘老庄和苏北灌溉总渠以北两大片区扶贫开发，全面完成脱贫奔小康工程的各项任务。

切实加强社会治理。积极构建现代化立体化社会治安防控体系，完善“六位一体”社会治理决策体系，打造平安淮安升级版。推行“阳光警务”，推广城区网格化管理、农村1公里警务圈管理模式，实现社区警务与社区治理高度融合。加快人口基础信息数据库建设，引导社会组织参与社会治理，深化政社互动和社区治理创新。开展矛盾纠纷排查化解，进一步规范信访秩序，提升应急处理突发事件的能力。强化党政同责、一岗双责、齐抓共管，切实筑牢安全生产防线。

各位代表，今年还有一项重要工作，就是研究编制“十三五”规划。规划编制将突出科学性、导向性和可行性，充分发扬民主，凝聚各方智慧，努力编制一个适应时代要求、符合淮安实际、反映群众意愿的“十三五”规划，为加快实现“两大目标”提供科学指导。

三、切实加强政府自身建设

经济发展进入新常态，对政府的施政能力提出了新要求。我们将始终坚持依法行政，加快转变政府职能，着力打造人民满意的法治型、服务型、效能型政府，努力以新常态下的新作为推动经济社会的新发展。

着力提高发展本领。积极转变发展理念，全面认识新常态下经济运行的新特征新趋势，切实把握大势、理清思路、精准施策，在全力稳增长的同时，顺势把结构优化好、把动力转换好、把风险防范好，牢牢把握科学发展的主动权。不断改进工作方式，深入研究国家和省新出台的一系列政策举措，积极探索破解融资、用地、招商等难题的有效路径，加快将政府工作的着力点转向更多地依靠提高效能和创新服务来优化发展环境。着力增强公共服务，主动适应公共需求总量的快速增长和结构的深刻变化，大力推进基本公共服务均等化，加快完善公共服务网络，进一步创新公共服务方式，积极构建多元化的公共服务供给体系。

深入推进依法行政。坚持法定职责必须为、法无授权不可为，以法治思维和法治方式做好改革发展稳定各项工作。健全依法决策机制，实行政府法律顾问制度，成立法制专家咨询委员会，在制定重大规划、出台重大政策和安排重大政府投资项目的过程中，严格遵循公众参与、专家论证、风险评估、合法性审查、集体讨论研究的决策程序。依法加强财政预算管理和政府合同审查备案工作，切实防范政府债务风险。深化行政执法体制改革，建立健全行政裁量权基准制度，全面落实行政执法责任制，积极推进行政执法与刑事司法的良性互动。完善重大事项向人大报告制度，落实政治协商“三在前、三在先”要求，重视司法监督、社会监督和舆论监督，全面推进政务公开，让行政权力在阳光下运行。

不断改进工作作风。切实巩固党的群众路线教育实践活动成果，健全落实中央八项规定精神的长效机制，持之以恒改进作风。完善联系服务群众工作机制，真心倾听群众呼声，及时回应群众关切，着力解决群众反映的突出问题。优化发展软环境，严格落实公开承诺、首问负责、限时办结和责任追究制度，加强督查和问责，坚决整治不作为和乱作为现象。进一步提升执行力，职责范围内的工作不折不扣完成，研究确定的任务千方百计落实，真正做到铁肩担责任、一心谋发展。

切实加强廉政建设。认真落实党风廉政建设责任制，全面推进惩治和预防腐败体系建设，着力营造风清气正的政务环境。加强政府部门权力制约，强化内部流程控制，构建监察、司法、审计等联动的行政权力监督机制，从源头上预防和减少腐败现象的发生。厉行勤俭节约，确保“三公”经费零增长。坚持从严要求，以零容忍的态度惩治腐败，努力建设勤勉尽责、秉公用权、清正廉洁的公务员队伍。

各位代表，适应新常态、抢抓新机遇、再上新水平，责任重大，使命光荣。让我们紧密团结在以习近平同志为总书记的党中央周围，在省委省政府和市委的坚强领导下，进一步解放思想、开拓创新，团结协作、真抓实干，为把周总理家乡建设得更加美好而努力奋斗!
